# Pulmonary Diseases

**Published:** 1894-02-03

**Authors:** 


### MEDICAL PROGRESS.

### PULMONARY DISEASES.

### PNEUMONIA.

*Pathology.*—Clinical experience has led us to differentiate between inflammations of the lungs due to or associated with different diseases, and the researches of Wassermann[1] in Professor Koch's Institute confirm this view. Pneumonia may be set up by Fränkel's diplobacillus, Pfeiffer's influenza bacillus, Friedländer's bacillus, the streptococcus, and the staphylococcus. An examination of the sputum from the deeper parts of the lungs enables us to determine the precise causative agent in any case. Thus (1) the streptococcus pneumoniæ Weichselbaum found in 21 cases in 129 pneumonias, and the author also found this microbe in two cases. Clinically there was difficulty in differentiating this from tuberculosis. (2) Pfeiffer's bacillus, found in cases of influenza pneumonias, may be of value in diagnosis in sporadic cases. Clinically, the sputum is not rusty, but frothy and purulent, the temperature curve irregular, and defervescence takes place by lysis.

Leucocytosis—a marked increase in the percentage of white blood corpuscles above the normal limit (8,000 per cubic millimetre)—has long been recognised as associated with certain physiological and pathological conditions. Dr. J. Ewing[2] has investigated the relations of leucocytosis to pneumonia. He first summarises the extent of our present clinical knowledge on this question as follows:—

During digestion and in pregnancy there is a moderate increase in the number of leucocytes.

In many pathological conditions, either cachectic or febrile, a constant and considerable leucocytosis has been demonstrated.

The principal cachectic conditions producing leucocytosis are three in number: 1. The acute anæmia following hæmorrhage. 2. The chronic anæmia of diseases of the blood and of malignant neoplasms. 3. The condition of the blood before death, giving an "ante-mortem" leucocytosis.

Finally, there is the leucocytosis of inflammatory conditions, which is a regular accompaniment of many febrile diseases. Of these may be mentioned lobar pneumonia, inflammation of serous membranes, meningitis, septicæmia, acute articular rheumatism, scarlatina, diphtheria. Measles, typhoid fever, and pulmonary tuberculosis have been shown to be important exceptions in this class. In typhoid fever, the number of leucocytes is regularly subnormal, while in phthisis it is usually inconsiderable.

Ewing states that the leucocytosis of lobar pneumonia has received more attention than that of any other febrile condition. Its course during the disease has been carefully followed in sixteen cases recently reported by Laehr. Its relation has been noted to the height of the fever, to the extent of the pulmonary lesion, to the amount of poison generated by the disease, and to the reaction of the system. Some have claimed that the degree of leucocytosis follows exactly the extent of the local lesion; others, that it is regulated by the amount of poison generated, and by the character of the systemic reaction excited by the disease. Its relation also to prognosis has been considered, and an attempt made to base a favourable prognosis on a high degree of leucocytosis. Von Jaksch[3] has observed that cases of lobar pneumonia unaccompanied by leucocytosis are of unfavourable prognosis. Laehr[4] reports a case in which a relapse was heralded by a recurrence of leucocytosis after its disappearance with the crisis. Laehr and Rieder believe that the examination of the blood is of great value in the diagnosis between lobar pneumonia and typhoid fever. The number of leucocytes in the blood in phthisis has been found so variable that no definite rules have been established as to the use of the blood count in distinguishing this disease from pneumonia.

In order to test the truth of the statement that a large increase in the number of leucocytes in the blood offers a favourable prognosis in lobar pneumonia, the writer examined the blood in 101 cases of this disease occurring in Roosevelt Hospital during the first five months of the year 1893.

From these investigations, Ewing concludes that: (1) In most cases of lobar pneumonia there is a marked leucocytosis. (2) The leucocytosis of lobar pneumonia may be absent or inconsiderable—(*a*) in very mild cases; (*b*) in very severe cases in which the reaction of the system is slight. (3) The degree of leucocytosis in pneumonia is proportional, on the average, to the extent of the local lesion, but it follows much more exactly the grade of systemic reaction to the poison generated. (4) In cases of acute tubercular inflammation of the lung, clinically resembling lobar pneumonia, there was no leucocytosis. (5) In pulmonary phthisis complicated by suppurating cavities, exudative pneumonia, pleurisy, or chronic anæmia, there was usually a moderate leucocytosis. (6) In typhoid and typhus fevers there was no leucocytosis. (7) In empyema and actinomycosis of the lung there was considerable leucocytosis. (8) In obscure forms of lobar pneumonia, in which ordinary physical signs and rational symptoms are insufficient for diagnosis, the examination of the blood may give useful evidence. (9) Well-marked leucocytosis is a valuable aid in the differential diagnosis between lobar pneumonia and typhoid or typhus fevers. (10) In acute apical lesions the absence of leucocytosis is decisive evidence in favour of tuberculosis, except when dealing with a lobar pneumonia which is very mild, or whose course is asthenic. (11) The examination of the blood is of no value in the diagnosis between lobar pneumonia and empyema or actinomycosis of the lung. (12) Well-marked leucocytosis in lobar pneumonia, while in itself a favourable sign, does not assure that the disease will pursue a favourable course, but indicates usually a severe infection. (13) A moderately low degree of leucocytosis in severe cases of lobar pneumonia is an extremely unfavourable sign. (14) In severe cases of lobar pneumonia absence of leucocytosis indicates, with rare exceptions, that the disease will prove fatal. (45) Most cases of lobar pneumonia in which the lesion extends to the pericardium and peritonæum are attended with slight leucocytosis.

*Treatment.*—ACONITE.—Arnot Spence, M.D.,[5] summarizes the results obtained in the treatment of 228 cases of pneumonia treated on the service of Dr. J. H. Ripley at St. Francis Hospital, New York, on the following plan in which it will be seen that the remedy relied on chiefly was aconite. To begin with, Dr. Ripley has the bowels freely moved by five grains of calomel triturated with sugar of milk; if the patient was too weak for this a gentle laxative was employed. The bowels having acted the following mixture was given, ℥ss., every two hours: ℞ Tr. aconiti ♏xxiv., tr. op. camph. ʒii., liq. ammon. acet., syr. Zingiberis aa. ʒjs., aquæ ad ℥vi., M. sign. ℥ss. every two hours. The aconite was given primarily for its effect on the heart and blood vessels, and thus on the inflamed lung, and secondarily to reduce the temperature. It produces a quieting and slowing of the irritable and rapid heart, and a reduction of the arterial pressure, thereby lessening the amount of blood in the congested lung. It controls the temperature within a safe range and prevents the marked exacerbations so common in typical cases of the disease. In strong and full-blooded patients *veratrum viride* was substituted for the aconite in this mixture and used in about the same dose.

Throughout the administration of this mixture the heart was most carefully watched to note the effects of the drugs, and all things being favourable it was continued until the fall in temperature denoted the crisis of the disease. When further stimulation seemed necessary whiskey was freely given, but never unless it was indicated. The administration of alcoholic stimulants all through the disease, as is frequently done, is a practice capable of doing great harm.

Morphine was given when necessary to overcome sleeplessness from any cause, but the free or constant use of morphine was never resorted to, for it marks the signs of dangerous symptoms, and moreover, by diminishing the number of respirations, interferes with the proper oxygenation of the blood.

If, notwithstanding the use of the pneumonia mixture, the temperature rose and remained above 104 degrees the cold half pack was resorted to in most cases. The antipyretic drugs were given only on rare occasions, and then never by order of the visiting physician, who has always strenuously opposed their use. Dr. Spence says that it is unreasonable to expect that a few hours remission of fever, the result of any of these depressing antipyretics, can better the condition of the patient or even compensate for the bad effect the drug leaves on the heart; the indiscriminate employment of these antipyretic drugs in every case of fever or rise of temperature cannot be too severely condemned.

The only attempt at local applications was the wearing of an oilskin jacket next the skin, worn throughout the treatment. The diet in all cases was limited, until convalescence set in, to milk, meal broths, soups, and farinaceous foods.

The 228 cases treated by the method can be classified as follows:—

| | Cases. | Deaths. | Mortality. Per cent. |
|---|---|---|---|
| Croupous pneumonia, uncomplicated | 125 | 18 | 14·4 |
| Croupous pneumonia, complicated... | 76 | 25 | 32·8 |
| Catarrhal pneumonia, uncomplicated | 25 | 5 | 20·0 |
| Catarrhal pneumonia, complicated... | 2 | 0 | 0·0 |
| Total number ............ | 228 | 48 | 21·05 |

The author classed the cases as uncomplicated so long as the process was confined to one lung, and no other complication existed.

The author from various analyses of his results in reference to age of patients, the nature of complications, &c., draws interesting and valuable conclusions, for which reference may be made to the original article.

Digitalis.—The credit of initiating the now prevalent method of treating cropous pneumonias with large doses of digitalis, belongs to Pétresco, and his paper[6] giving a *résumé* of the work of various Continental observers, together with a table of the effect of this treatment upon fifteen new cases, is authoritative. Pétresco claims that large doses of digitalis absorb the pneumonia, producing a fall of temperature and pulse rate, and curtailing the attack by two or three days, and most observers agree with this statement. The treatment consists in the daily administration of sixty to ninety grains of digitalis leaves in the form of an infusion, continued for two or three days, at the end of which time the effect of the digitalis is usually evident. The temperature falls 3 to 5 deg.; pain and dyspnœa disappear, and the pulse diminishes to a half or a third of its previous rate. Pneumonic patients are very tolerant of the drug. Toxic effects are rarely if ever observed. Age is no contra-indication, thus Hoeffel gave large doses to children of ten and to patients over sixty. Huchard does not regard albuminuria as a contra-indication, and states, on the authority of Lafone, that digitalis is not excreted by the kidneys. The author believes that the beneficial effect of the drug is due to its action on the heart and blood vessels. During the last twelve years Pétresco has treated 1,061 cases by this method with marked success. The mortality is given by Antonin, on an analysis of 577 cases, at 1·41 per cent., and by Constantinesco, on the observation of 816 cases, at 2·06 per cent.

M. Bellotti,[7] in a paper, a continuation of one published about a year ago in reference to the same subject, deals with the treatment of croupous pneumonia, due to Fränkel's diplococcus, and, as the result of extensive observations, concludes: (1) That all cases derive great benefit from the digitalis; (2) that the doses must be very large, as owing to the gastric catarrh and diminution of H.Cl. in the gastric juice, a large proportion of the drug is not absorbed; (3) that some of the active principles of digitalis have an elective affinity for a special action on the toxins of pneum[illegible]nia, this action occurring mainly in the liver.

Other cardiac tonics, *e.g.*, Strophanthus and Adonis, have been used in substitution for digitalis by Lazzaro[8]. He regards the beneficial action of digitalis as being due to its reinforcing the systole of the heart, and so enabling it to surmount the circulatory obstacle set up by the congested lung, and then to do away with the œdema. He found that strophanthus and adonis had a more prompt action, and are more tolerated than digitalis. In two typical cases he gave daily 1·5 gramme of tr. strophanthi, and 6· gramme of adonis respectively. He is opposed to the routine exhibition of cardiac tonics in pneumonia, considering that they should be reserved for cases in which cardiac failure threatens.

Chloride of Calcium, advocated by Dr. A. Crombie[9], has been found very beneficial in pneumonia in the hands of Mr. J. Couldrey. "In two especially severe cases, in which under the old treatment a fatal result might have been expected," recovery took place. This observer gives an instructive chart of one case in which the treatment, commenced on the second or third day (December 23rd), speedily reduced the temperature and caused the lung to clear up. We may recall Crombie's observations in 22 cases of fibrinous pneumonia in which chloride of calcium, in dose of 0·3-1 gramme every four hours, gave such rapid results, and which he attributed to its action in neutralising the toxins.

[1] Deut. Med. Woch, November 23rd, 1893; Brit. Med. Journ., December 16th, 1893. [2] New York Med. Journ., December 16, 1893. [3] Clinical Diagnosis. [4] Weber das Auftreten v. Lene, b. cr. ln., Deut. Med. Woch, 36, 37, 1893. [5] Med. Rec,, July 1st, 1893. [6] Rev. de Méd., March, 1893; Med. Chron., May, 1893. [7] Gazz. Degli. Osp., July 22nd, 1893; Brit. Med. Journ., September 23rd. [8] Arch. di Farmac e Terap., June 15th; Brit. Med. Journ., September 16th, 1893. [9] Practr., April, 1893.

